# Tsetse Fly (*G*.*f*. *fuscipes*) Distribution in the Lake Victoria Basin of Uganda

**DOI:** 10.1371/journal.pntd.0003705

**Published:** 2015-04-15

**Authors:** Mugenyi Albert, Nicola A Wardrop, Peter M Atkinson, Steve J Torr, Susan C Welburn

**Affiliations:** 1 Coordinating Office for Control of Trypanosomiasis in Uganda, Kampala, Uganda; 2 Division of Infection and Pathway Medicine & Centre for Infectious Diseases, School of Biomedical Sciences, College of Medicine & Veterinary Medicine, The University of Edinburgh, Edinburgh, Scotland; 3 Geography and Environment, University of Southampton, Southampton, United Kingdom; 4 Department of Physical Geography, Faculty of Geosciences, University of Utrecht, Utrecht, Netherlands; 5 Liverpool School of Tropical Medicine, Liverpool, United Kingdom; 6 Warwick Medical School, University of Warwick, Coventry, United Kingdom; Liverpool School of Tropical Medicine, UNITED KINGDOM

## Abstract

Tsetse flies transmit trypanosomes, the causative agent of human and animal African trypanosomiasis. The tsetse vector is extensively distributed across sub-Saharan Africa. Trypanosomiasis maintenance is determined by the interrelationship of three elements: vertebrate host, parasite and the vector responsible for transmission. Mapping the distribution and abundance of tsetse flies assists in predicting trypanosomiasis distributions and developing rational strategies for disease and vector control. Given scarce resources to carry out regular full scale field tsetse surveys to up-date existing tsetse maps, there is a need to devise inexpensive means for regularly obtaining dependable area-wide tsetse data to guide control activities. In this study we used spatial epidemiological modelling techniques (logistic regression) involving 5000 field-based tsetse-data (*G*. *f*. *fuscipes*) points over an area of 40,000 km^2^, with satellite-derived environmental surrogates composed of precipitation, temperature, land cover, normalised difference vegetation index (NDVI) and elevation at the sub-national level. We used these extensive tsetse data to analyse the relationships between presence of tsetse (*G*. *f*. *fuscipes*) and environmental variables. The strength of the results was enhanced through the application of a spatial autologistic regression model (SARM). Using the SARM we showed that the probability of tsetse presence increased with proportion of forest cover and riverine vegetation. The key outputs are a predictive tsetse distribution map for the Lake Victoria basin of Uganda and an improved understanding of the association between tsetse presence and environmental variables. The predicted spatial distribution of tsetse in the Lake Victoria basin of Uganda will provide significant new information to assist with the spatial targeting of tsetse and trypanosomiasis control.

## Author Summary

Trypanosomiasis is a vector-borne disease transmitted to both humans and animals by the tsetse fly. The tsetse vector is distributed across sub-Saharan Africa. Trypanosomiasis maintenance is determined by the interrelationship of three elements: vertebrate host, parasite and the vector responsible for transmission. Mapping the distribution and abundance of tsetse flies assists in predicting trypanosomiasis distributions and developing rational strategies for disease and vector control. This study makes available dependable tsetse fly distribution data (maps) for use by decision makers. The approach makes use of modelling techniques involving limited field-sampled tsetse data points distributed across an area of approximately 40,000km^2^ within the Lake Victoria basin of Uganda. Precipitation, temperature, landcover, normalised difference vegetation index (NDVI, a measure of the amount of green vegetation) and elevation data were used as environmental covariates. We used logistic regression to analyse the relationships between presence of tsetse and the environmental covariates. The results indicated that tsetse are more likely to be present in areas with a greater proportion of riverine vegetation and forest cover. The key outputs are a predicted tsetse distribution map for the Lake Victoria basin of Uganda and an increased understanding of the association between tsetse presence and environmental variables. This will provide a vital resource for the planning and spatial targeting of future tsetse control activities.

## Introduction

Tsetse flies are responsible for the transmission of human African trypanosomiasis (HAT), also known as sleeping sickness and its animal form (nagana). Trypanosomiasis occurs in 38 sub-Saharan African countries with an average of 15,000 human cases reported annually (period 2000–2012 [1]), and 70 million people at risk of contracting the infection [2]. Uganda reports approximately 500 cases of sleeping sickness annually [1], and it is the only country reporting the presence of both forms of HAT: the gambiense form in the north-west and the rhodesiense form in the south-east and, more recently, in the centre of the country [3, 4]. Animal trypanosomosis presents major constraints to livestock production among many livestock keeping communities in Africa. The disease is widely reported in Uganda [5], and the removal of African animal trypanosomiasis (AAT) could generate direct economic benefits in the region of 400 million US$ in a 20-year period [6].

*Glossina fuscipes fuscipes* is known to be present in several parts of Uganda, with its geographical extent stretching from Lake Victoria’s shores through central Uganda up to the West Nile region. In addition, *G*. *f*. *fuscipes* is assumed to be present around Lakes Albert, Edward and George in western Uganda. The islands of Kalangala and Buvuma located within Lake Victoria have also been identified as having *G*. *f*. *fuscipes* [7].

The major drivers of tsetse fly habitation are generally known to be temperature, humidity, rainfall, vegetation and presence of host animals [8, 9, 10]. This implies that tsetse flies are found in ecologically suitable habitats, represented through a set of conditioning environmental variables. Such variables determine: feeding behaviour; infection rates; fly movements; fly density; species-diversity; and fly reproduction [10]. Therefore, spatial information on such environmental variables can be helpful in predicting the relative distribution of tsetse flies in an area.

Tsetse distribution maps are crucial in the control and management of human and animal trypanosomiasis in affected areas [11, 12]. Accurate maps should ideally be based on high precision fly data derived from field investigations. In the absence of such data, tsetse distribution maps may be constructed using partial district-level entomological reports, existing publications, sector reports and modelled environmental covariates. Given scarce resources to carry out regular field tsetse surveys, there is a need to devise inexpensive means for periodically obtaining reliable large area and high precision tsetse information across target areas. A potential solution is provided by spatial statistical modelling (e.g., spatial regression analysis) using tsetse presence or abundance data acquired from field survey and fine spatial resolution satellite-generated environmental variables.

Regression is a statistical tool used to quantify the association between an outcome measure and predictor variables [13]. Logistic regression, in particular, is commonly used to explain or predict a binary variable response using a set of predictor variables or covariates [14]. This approach has been used in the predictive mapping of various vectors and associated vector-borne diseases including malaria and Rift valley fever, with broad applications in environmental disease risk [15]. The use of GIS and temporal Fourier-processed surrogates for vegetation, temperature and rainfall derived from satellite sensor data in predicting tsetse distributions has been investigated with significant utility [16]. Further use of GIS and remote sensing in attempting to explain tsetse vector distributions is described in Rogers *et al*.[17, 18] and Wint *et al*. [19, 20, 21].

Wint and Rogers [19, 21], at a spatial resolution of 5 km, predicted tsetse presence at the continental level using logistic regression, targeting 23 tsetse sub-species from the three major species groups (*Fusca*, *Palpalis* and *Morsitan*). The process involved fitting statistical regression models between tsetse data and remotely sensed predictor variables. The tsetse data used were derived from the Ford and Katondo tsetse maps [22, 23], through systematic extraction of 12,000 points across the entire continent. Predictor variables included; NDVI, surface temperature, middle-infrared reflectance, vapour pressure deficit and surface rainfall [19, 21].

Wint [20], in an effort to provide more accurate tsetse maps, derived sub-continental tsetse fly distribution maps at a spatial resolution of 1 km for East Africa (Uganda) and selected parts of some countries in West Africa. This approach made use of; (i) modified Ford & Katondo presence/absence maps, (ii) 5 km-continental tsetse predictions in 2000, (iii) 17,000 data points extracted for East Africa and satellite-derived data. According to these maps, Uganda is approximately 80% tsetse infested. Although an improvement from the Wint continental version [19, 21], these sub-continental tsetse distribution maps are associated with low precision. The lack of up-to-date field data on tsetse is a key concern, while the absence of land cover data as a predictor, which is known to be important in determining tsetse distributions, is another.

In Uganda, there is a need to produce dependable and up-to-date tsetse distribution information, preferably at sub-national level, to support decision-making and improved planning of tsetse control interventions. Relatively few studies have used recently gathered data from traps. The purpose of this study was to quantify the relationships between tsetse presence/absence and external factors in the study area and also to predict the spatial distribution of *G*. *f*. *fuscipes* in the Lake Victoria basin of Uganda.

## Methods

The study area is predominantly a lake basin stretching for approximately 50 to 100 km from the Lake Victoria shoreline in Uganda. This region is characterized by high annual rainfall (1000–1500 mm) with two distinct rainfall peaks in April and November. Tsetse data were obtained from a systematic entomological survey conducted from May to June, 2010, to ascertain tsetse presence and abundance. Biconical traps [24, 25] were used to capture tsetse flies during the survey. Five thousand geo-referenced tsetse trap sites were spread uniformly over a ground area of approximately 40,000km^2^ within the target region [26]. Trapping at each site lasted 72 hours and was conducted by teams led by district entomologists. Single collection was made at the end of this 72 hour period. The parameters recorded in the entomological survey sheet included: trap code, latitude, longitude, altitude, vegetation type around the trap site, start date and time, end date and time, species trapped, number of females, males and flies of un-identified sex, and number of other biting insects. Data were collated and entered into a database. These tsetse data were used as the dependent variable in the regression modelling, while all other variables were used as independent variables.

Several covariates (Table 1) were used in the analysis, based on an understanding of the factors important for tsetse reproduction and survival [11, 27, 28]. These included; (i) land cover, (ii) temperature, (iii) normalised difference vegetation index (NDVI), (iv) elevation, and (v) rainfall. The land cover data were extracted from the fine spatial resolution, multi-purpose land cover dataset GlobCover for 2009 [29]. This global land cover series is described by a legend of 22 core land cover categories in total. The region under study contained only 19 of the 22 classes presented. Land cover variables used in the analysis were estimated through the creation of buffers of 1000 m (catchment) around each entomological tsetse survey point. Within each buffer, area percentages of the different land cover types were computed and used as the set of land cover predictor variables. NDVI, as a measure of vegetation cover or biomass production, was derived from the National Oceanic and Atmospheric Administration (NOAA) Global Inventory Monitoring and Modelling Studies group (GIMMS) dataset [29]. The temperature and precipitation data used were obtained as interpolated raster data at a spatial resolution of 30 arc-seconds from the *WorldClim—Global Climate Data* facility [29]. Elevation data were obtained from the Shuttle Radar Topography Mission (SRTM).

**Table 1:** Covariates used in the analyses of tsetse fly distribution and abundance including their observed maximum and minimum values in the training dataset.

Code	Name	Max value	Min value
**Meteorological data surrogates**	*Rainfall(mm)*		
	Monthly total-April	331	123
	Monthly total—May	339	79
	Monthly total—June	154	21
**Meteorological data surrogates**	*Temperature (* ^*0*^ *C)*		
	Max Temp (April)	29.7	25.7
	Mean temp (April)	24.0	20.4
	Min Temp (April)	18.4	15.2
	Max Temp (May)	28.8	26.0
	Mean Temp (May)	23.5	19.8
	Min Temp (May)	18.2	14.6
	Max Temp (June)	28.6	22.0
	Mean Temp (June)	23.2	19.6
	Min Temp (June)	17.8	13.5
**Vegetation surrogates**	*Normalised difference vegetation index (NDVI)*		
	NDVI-1 (April)	0.90	0.02
	NDVI-2 (May)	0.90	0.01
	NDVI-3 (June)	0.90	0.00
**Altitude**	*Elevation (m)*	1034	1412
**Land cover**	*Land cover* (22 Classes)	n/a	

Tsetse survey count data were transformed to a binary variable representing tsetse fly presence or absence (0, 1). Presence of tsetse flies was represented by a “1” while absence was represented by “0”. Preliminary visualisation of the geographical distribution of tsetse presence was carried out using the ArcMap10 GIS software (ESRI, Redlands). Exploratory analysis was performed as a means to check for outliers, and aspects of homogeneity, normality and collinearity within the predictor variables.

A forward step-wise approach was applied to select the final multivariate logistic regression model. Covariates were added one after the other cumulatively and were retained if they retained statistical significance (*p* < 0.05). Estimated multivariate regression model coefficients were compared with those obtained at the univariate analysis stage to ascertain the consistency of final covariates in influencing the outcome variable.

A residual variogram was constructed to assess the presence of spatial autocorrelation in the model residuals. Autologistic regression was applied to account for the residual spatial autocorrelation [30, 31, 32, 33, 34, 35]. This process involves the introduction of a new explanatory variable (*autocovariate*). Autologistic regression involving several covariates is determined using the formula;

$$\ln\frac{\pi }{1-\pi_{i}}=\alpha +\beta s(y_{i})+\sum_{k}y_{k}x_{ki}+\varepsilon_{i}$$ 1

Where;

***ɑ*** is the model intercept

***β*** is the coefficient that relates to the autocovariate

***s(y***_***i***_ ***)*** is the *autocovariate* and is a function that summarises the *y*-values in the neighbourhood of ***i*.** It is calculated from the observed data only once and used throughout.

***γ***_***k***_ are the coefficients relating to the *k* different environmental covariates

***x***_***ki***_ are the ***k*** different environmental covariates at location ***i*.*Ԑ***_***i***_ is the error

The spatial autocorrelation was quantified by the Global Morans’s *I* index as extending up to a distance of 20 km [31, 32]. Thus, a spatial range of 20 km was used for the calculation of the autocovariate.

Receiver operating characteristic (ROC) curves were generated to evaluate model performance based on suggested cut-off points. Sensitivity and specificity were used to assess the predictive ability of the model. The area under the ROC curve (AUC) was calculated to provide an assessment of how accurately the model can classify the study area into tsetse presence and absence [13, 36]. Spatial prediction was carried out using the final multivariate model parameters, along with spatially continuous covariate datasets, to enable visualisation of predicted probability of occurrence for both the sampled and unsampled locations. The unsampled locations were represented on a regular grid and the predictions were used to produce continuous surface maps. The probabilities were derived from the regression equation in which the linear predictor was transformed using the logit function into a value between 0 and 1. Values close to ‘0’ represent a high probability of tsetse absence while ‘1’ represents a high probability of tsetse presence. All analyses were performed using the software R, version: Rstudio2011, with additional packages; *geoR*, *gstat*, *MASS* and *spdep*.

## Results

**Fig 1:**
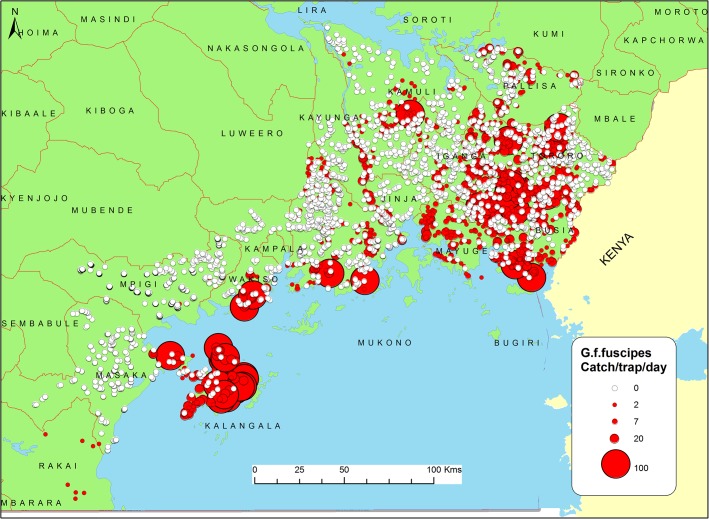
Binary map of tsetse presence and absence, illustrating the extensive nature of the field survey. It shows the tsetse survey outcome categorised as ‘present’ and ‘absent’. All tsetse traps which had tsetse flies where categorised as ‘present’ while those without tsetse flies where categorised as ‘absent’. G.f.fuscipes were captured in only 28.8% of the sampling sites (“present”). This implies that 71.2% of the trapping sites registered zero catches (“absent”). A total of 14,899 G.f.fuscipes flies (females = 7138, males = 7271 and 108 unidentified sex) were caught during the survey.

A map of tsetse abundance based on the tsetse sampling points is presented in Fig 1. These data indicate spatially heterogeneous distributions, with high tsetse abundance particularly in the Kalangala islands, along the river Nile, and in the south eastern regions of the study area.

In an initial univariate logistic regression stage, 44% of the land cover variables had a statistically significant association with tsetse presence-absence (*p*<0.05). Covariates; cropland, forest, riverine vegetation, woody vegetation, NDVI, elevation, temperature and rainfall were all positively correlated (*p<*0.05, Odds ratio (OR)*>*1), while savannah vegetation, herbaceous vegetation and built-up area were negatively correlated (*p<*0.05, OR<1) with tsetse presence.

Seven covariates were included in the multivariate logistic regression model. The significant covariates were; rainfall, elevation, temperature, cropland, savannah vegetation, forest, and riverine vegetation. Parameter estimates are given in Table 2. The presence of tsetse flies was negatively correlated with savannah vegetation, and positively correlated with the remainder of the model covariates. However, the covariates cropland, riverine vegetation, elevation and rainfall presented only very small positive associations, with wide confidence intervals.

**Table 2:** Multivariate regression: Variables used in logistic model fitting and their estimated parameters.

		Estimate	SE	*P*-Value	Odds Ratio	C.I (95%)
	Intercept	<0.001	3.084	P<0.05	<0.001	<0.001–0.001
1	Cropland	0.003	0.001	P<0.05	1.00	1.001–1.005
2	Savannah	- 0.007	0.001	P<0.05	0.99	0.991–0.995
3	Forest	0.254	0.042	P<0.05	1.29	1.190–1.403
4	Riverine vegetation	0.007	0.004	0.0501	1.01	1.000–1.014
5	Temperature	0.967	0.092	P<0.05	2.63	2.200–3.15
6	Elevation	0.006	0.001	P<0.05	1.01	1.004–1.008
7	Rainfall	0.020	0.001	P<0.05	1.02	1.017–1.023
AIC = 4925.7, DF = 4579						

The map of residuals and the residual variogram based on the multivariate logistic regression model revealed the existence of residual spatial autocorrelation. This situation is a problem as it violates the assumption of independence of residuals and can result in biased parameter estimates, leading to inflation of significance. Since non-spatial models fail to account for the autocorrelation effect, there was a need to apply a spatial model: in this case, autologistic regression [37, 38].

**Fig 2:**
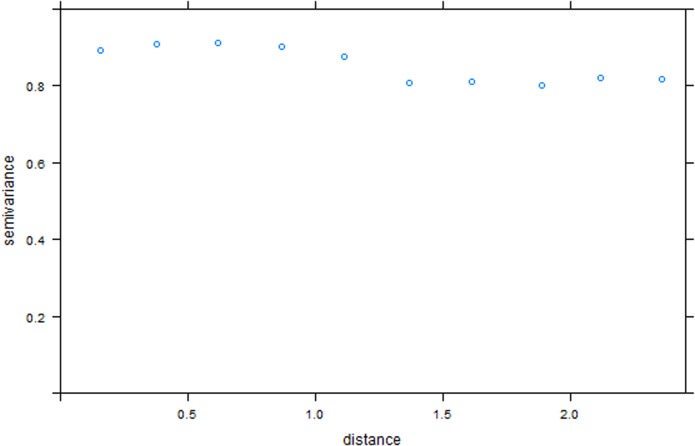
Spatial autocorrelation in the residuals from autologistic regression. Residual variogram of residuals from autologistic regression. This is evidence for reduced spatial autocorrelation in the residuals.

Autologistic regression was applied based on the seven significant variables obtained from the multivariate logistic model together with the computed autocovariate. The resultant statistics are presented in Table 3 and the residual variogram from the autologistic model is shown in Fig 2. The residual variograms for the two models were compared. The autologistic regression model reduced the spatial autocorrelation in the residuals compared to the multivariate logistic model.

**Table 3:** Autologistic regression model statistics.

Covariate	Estimate	SE	P_value	OR	C.I (2.5%)
Autocovariate	2.316	0.273	p< 0.05	765.09	451–1316
Forest	0.100	0.047	p< 0.05	1.105	1.010–1.214
Riverine vegetation	0.008	0.004	p< 0.05	1.008	0.999–1.016
Savannah vegetation	- 0.007	0.001	p< 0.05	0.993	0.991–0.996
Elevation	- 0.003	0.001	p< 0.05	0.997	0.995–0.999
Cropland	- 0.002	0.001	0.118	0.998	0.995–1.001
Rainfall	- 0.001	0.002	0.445	0.999	0.995–1.002
Temperature	- 0.018	0.110	0.871	0.982	0.790–1.219

In the autologistic model, forest (*p*<0.05, OR = 1.105) and riverine vegetation (*p*<0.05, OR = 1.008) were positively correlated with tsetse presence. Savannah vegetation (*p*<0.05, OR = 0.993) and elevation (*p*<0.05, OR = 0.997) were negatively correlated. These three land cover classes and elevation are, thus, considered to be important determinants of tsetse presence and absence in the study area. Cropland, temperature and rainfall failed to retain their significant association with tsetse presence (*p*>0.05) after accounting for spatial autocorrelation.

The Pearson *X* ^2^ test parameter and Deviance parameter were evaluated as measures of goodness-of-fit. These measures were statistically non-significant (Pearson *X* ^2^ = 4654, p = 0.196 (i.e p>0.05) and Deviance = 4890), indicating that the model fits the data appropriately and, therefore, could be used to predict probabilities of tsetse presence across the study area.

**Fig 3:**
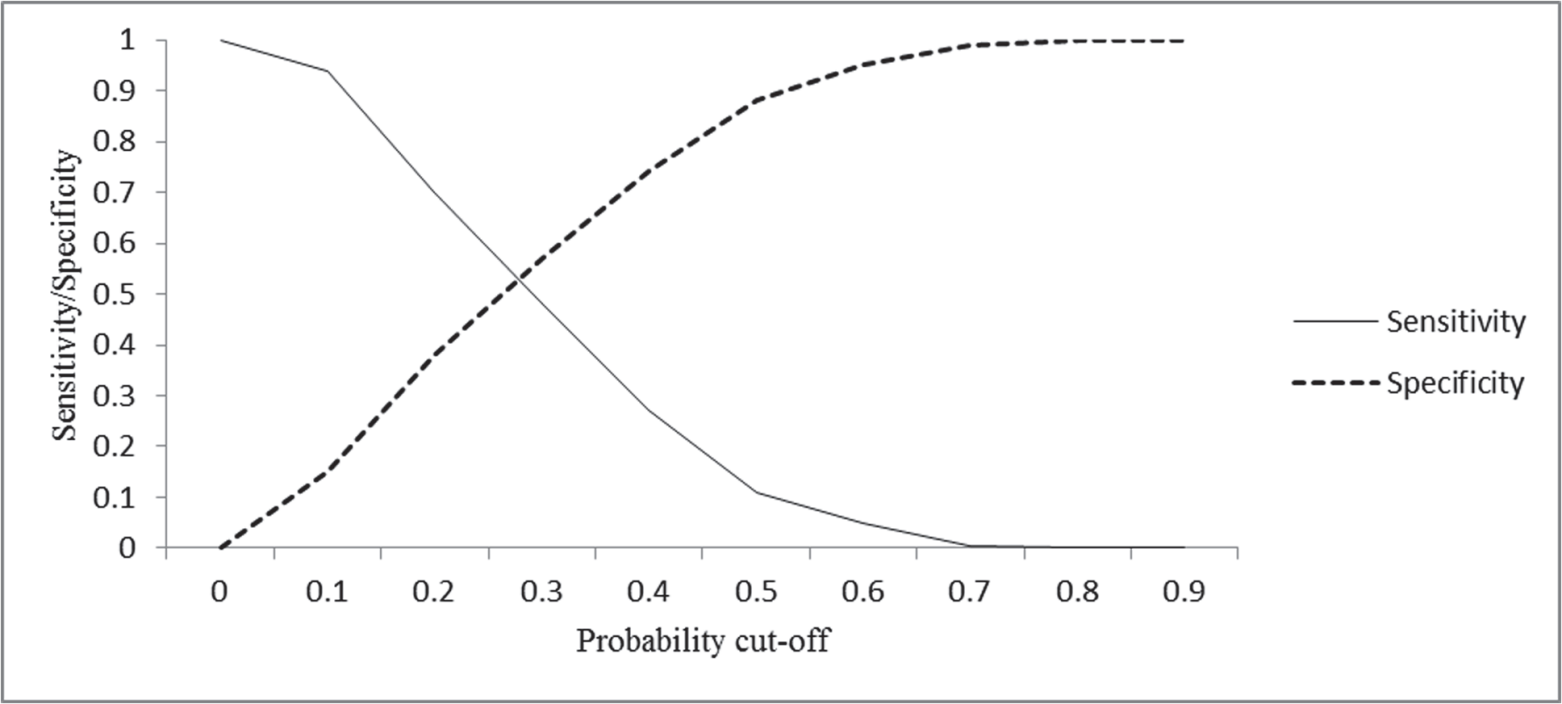
Use of two-graph receiver operating characteristic (ROC) curves. This is the plot of sensitivity and false positives (1-specificity) against expected probabilities and indicates that probability cut-off point is at 0.28, leading to a sensitivity and specificity of 53%. This is the threshold value for the prediction of tsetse presence where both specificity and specificity are maximised.

Model evaluation was conducted to assess prediction accuracy. The area under the curve (AUC) was computed as 72.7%, indicating adequate predictive ability. The plot of sensitivity and false positives (1-specificity) against expected probabilities (Fig 3) indicates a probability cut-off point of 0.28, leading to a sensitivity and specificity of 53%. This is the threshold value for the prediction of tsetse presence where both sensitivity and specificity are maximised, and can be used to classify areas as containing tsetse or not [39]. At a probability cutoff of 0.5, the sensitivity is 10% while specificity is 90% (Fig 3). This implies that at this cutoff approximately 90% of all the true positive cases (tsetse presence) will be missed. As the threshold increases, the sensitivity decreases and the specificity increases.

**Fig 4:**
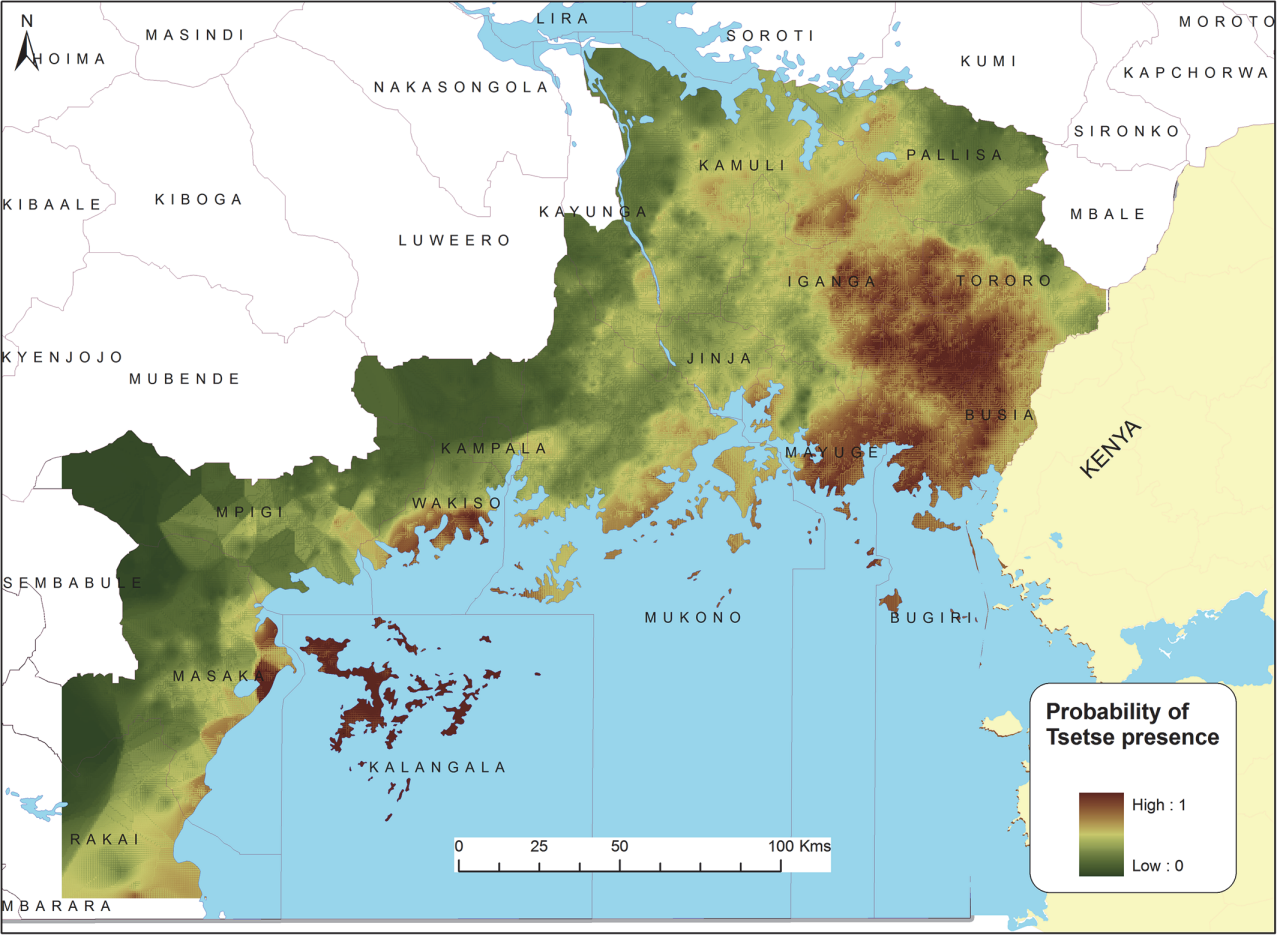
Predicted distribution of probabilities of G.f.fuscipes presence in the study area based on a logistic regression model.

**Fig 5:**
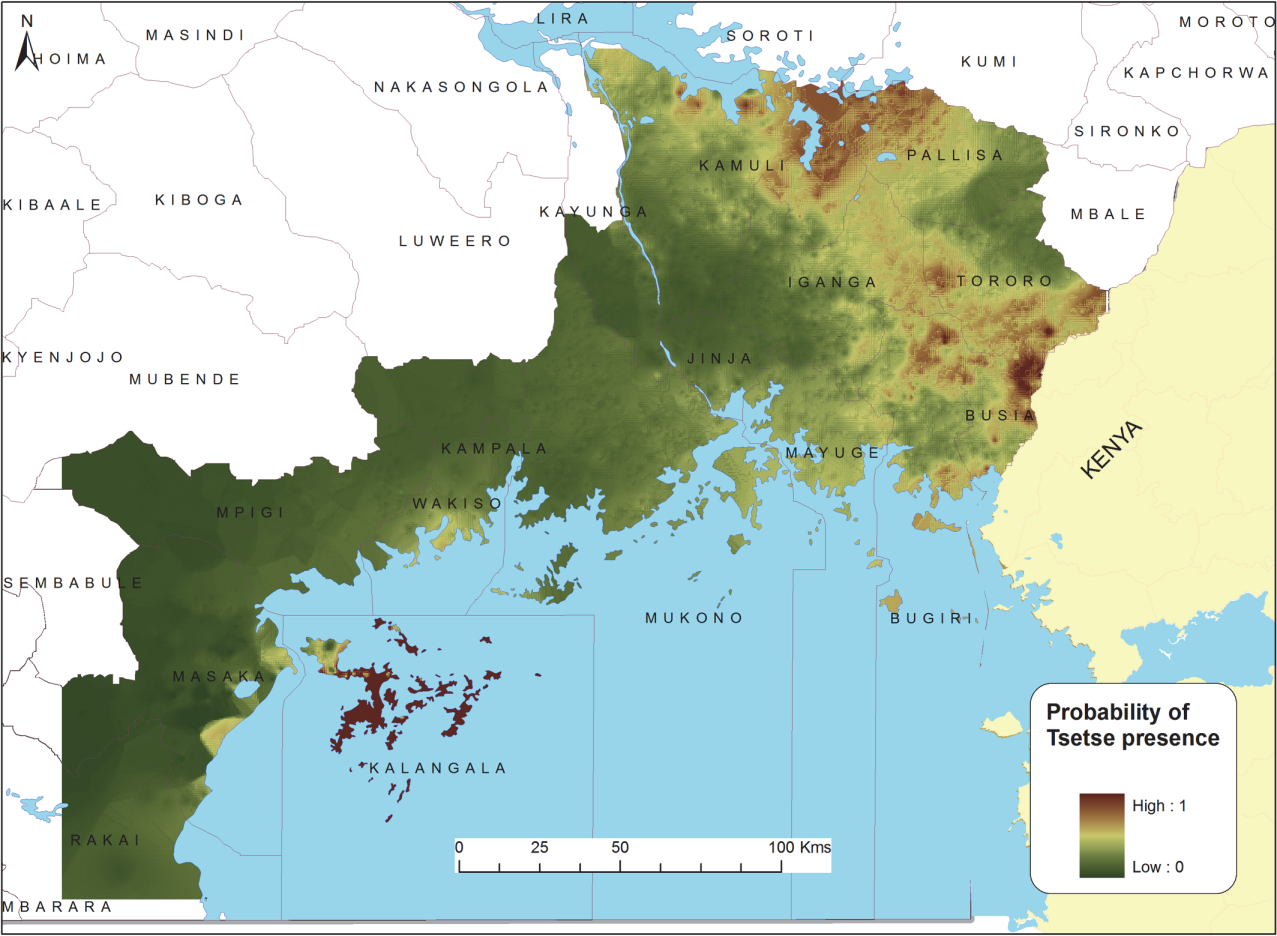
Predicted distribution of probabilities of G.f.fuscipes presence in the study area based on an autologistic regression model.

Fig 4 shows the predicted probability of tsetse presence across the study area, based on the multivariate logistic regression model (non-spatial model), while Fig 5 shows the predicted probability of tsetse presence across the study area, based on the autologistic regression model (spatial model). The two models identify areas of scaled potential tsetse fly risk with estimated probabilities of tsetse presence ranging from 0 to 1. The outcome reflects the presence of a clear tsetse infestation corridor in the Eastern part of the study area. High probability of tsetse occurrence (predicted probability of occurrence > 75%) was predicted in the eastern sections of the study area close to the Kenya-Uganda border (Bugiri, Busia, Tororo Kaliro, Kamuli and Pallisa districts) as well as on islands located in Lake Victoria. Low probability of tsetse occurrence (below 20%) was predicted in the western and north-western parts of the Lake Victoria basin.

## Discussion

The primary objective of the study was to develop a predictive model that can reliably inform decision-makers about the spatial distribution of *G*.*f*.*fuscipes* in the target area of Uganda, based on entomological survey results and a set of environmental covariates. The research was intended to provide high precision, up-to-date, sub-national tsetse maps to guide control interventions. The tsetse presence and absence data (dependent variable) represent one of the most comprehensive tsetse datasets collected over such a large area and are fully geo-referenced.

At the univariate investigation stage, tsetse presence was found to be significantly (positively) associated with eight variables (cropland, woody vegetation, forest, riverine vegetation, NDVI, elevation, temperature and rainfall (*P*<0.05, OR>1)). Rainfed cropland, savannah vegetation and herbaceous vegetation demonstrated a negative association (p<0.05, OR<1). Temperature demonstrated the largest correlation with the outcome variable (*p*<0.05, OR = 2.61).

The multivariate logistic regression model established that the presence of tsetse was positively associated with temperature, elevation, rainfall and proportion of forest cover, riverine vegetation and cropland. Savannah vegetation was negatively correlated with the outcome. Temperature remained highly influential in determining tsetse presence in the multivariate model (p<0.05, OR = 2.63). Tsetse flies are very sensitive to environmental changes and ecological instability, and are found in ecologically suitable habitats which have the necessary temperature, humidity and vegetation cover [40]. *G*.*f*.*fuscipes*, as a riverine species of the *palpalis* group, thrives in zones with high humidity [41].

After accounting for spatial autocorrelation, the covariates temperature, rainfall, and cropland lost their statistical significance in influencing tsetse presence or absence. The use of a spatial autologistic regression enabled the detection of key environmental variables that are highly influential in positively determining tsetse presence and these were; forests and riverine vegetation. Savannah vegetation (p<0.05, OR = 0.993) and elevation (p<0.05, OR = 0.997) retained their negative association with tsetse presence. The discussion below is based entirely on the results from the spatial regression model (autologistic regression).

Tsetse presence was positively correlated with forest cover. These correlations are consistent with the known aspects of the fly’s ecology [10]. Tsetse (*G*.*f*.*fuscipes)* thrives in environmental conditions where the vegetation is not too dense such as to enable them to fly easily and spot the feeding host readily. In addition, tsetse presence was positively correlated with riverine landcover. *G*. *f*. *fuscipes* is ecologically considered a riverine species and is commonly found in zones of high humidity offered by the interaction between forest vegetation and water bodies. It is important to make use of data with a fine spatial resolution, especially when considering drainage systems, to enable the identification of small rivers and streams that may support riverine vegetation. The spatial resolution of the land cover data used may not have been detailed enough to enable small rivers to be detected.

Tsetse presence was negatively correlated with savannah vegetation. Such vegetation can be categorised as “humanised” or “disturbed” landscapes, and tsetse flies usually avoid disturbed habitats [40]. Additionally, the low humidity in savannah landscapes (due to less water and vegetation cover) is less suitable habitat for riverine tsetse flies. Tsetse presence was also negatively correlated with elevation. Such association has been detected in previous research [42]. Generally, elevation may influence the micro-climatic conditions or landcover variations of an area. However, the entire study area had limited height variation (1034 to 1412 m asl) and the model, thus, illustrates the lack of an altitudinal control on tsetse presence within this particular study area, as evidenced by the odds ratio which was close to unity. Water courses are located at lower elevations. Thus the altitude effect is bound to be influenced by proximity to existing waters courses.

Tsetse presence was not correlated with cropland (p>0.05). This association could be linked to its characteristic of being a completely humanised landscape. There is a tendency for tsetse flies to avoid such environments [40] due to removal of vegetative cover ideal for tsetse survival. However, following habitat degradation, *G*. *f*. *fuscipes* can take refuge in remnant tree cover (thickets), which may explain the presence of tsetse in cultivated fields during the survey [43]. Tsetse presence was not significantly correlated with rainfall. The entire study area had monthly total rainfall ranging from 34 to 339 mm with no significant spatial variation across the study area. Therefore, it is unlikely that precipitation would influence tsetse distributions.

Tsetse presence was not significantly correlated with temperature (p>0.05). Tsetse flies thrive in areas with mean annual temperatures between 19 and 30°C [44]. Temperatures below 19°C slow down tsetse activity and general physiology [44], while extreme low temperatures (below 15^°^C) increase fly mortality [45]. Tsetse are severely affected by high temperature conditions and once exposed to a temperature of more than 36°C tsetse will have a survival capacity of close to zero [46]. From the training data, the lowest temperature for the study area was recorded as 13.5°C, the mean temperature was 27°C and the maximum temperature was 29.7°C. Temperature variation was by about 4°C at most sites across the region. These temperature ranges were within the acceptable envelope for the fly and therefore had no specific consequences on fly availability in the study area.

Fitting the autologistic regression model permitted us to assess the influence of spatial autocorrelation on the probability of tsetse presence. The parameters for temperature, rainfall, and cropland appeared less important (not statistically significant) after accounting for the effect of forest cover, riverine vegetation, elevation and spatial dependence in the observations. The predictive outputs from the autologistic regression model are considered to be more reliable than those from the initial logistic regression model, as they account for spatial autocorrelation in the data by incorporating information from neighbouring locations. The autocovariate term captured part of the spatial pattern in the data observations, thus, providing a more robust estimation of the covariate effects after accounting for the spatial dependence in the observations. The autologistic regression predictive outputs should be considered to be the most important in terms of future planning of interventions. However, it should be noted that the predictive models did not predict tsetse presence along the river Nile and this may be due to the spatial resolution of the covariate data used (1 km) not allowing accurate representation of relatively small areas of suitable habitat.

Other methodological approaches can be used to deal with spatially autocorrelated data, such as model-based geostatistics [15], although the fitting of these models and subsequent spatial predictions are very demanding computationally. Future research will refine the spatial models presented in this paper using these computationally intensive methods. The present analysis provides much needed empirical data on tsetse distributions in south east Uganda, along with spatially continuous predicted outputs which will provide significant benefits for the planning of future interventions.

### Conclusion

Several tsetse sub-species have long been associated with the Lake Victoria basin. The location-specific entomological data gathered for this study provide further evidence of the extensive distribution of tsetse in the area. Using logistic and autologistic regression models coupled with extensive field survey entomological data and a set of environmental covariates, a tsetse distribution map for the lake basin was constructed. These regression models enabled the identification of the important environmental variables determining tsetse presence across the study area. Notably, the final model identified forests and riverine vegetation (positive) and savannah vegetation and elevation (negative) as the key covariates associated with tsetse presence in the study area. Knowledge of the influential factors and availability of detailed sub-national tsetse distribution maps offers a platform for making meaningful decisions when planning tsetse control interventions. The findings are based on data from Uganda, but the approach is certainly of much broader interest and application.
